# Peripheral Nerve Decellularisation Protocol for Allogeneic Transplantation: From Tissue Procurement to Banking

**DOI:** 10.3390/ijms26167937

**Published:** 2025-08-17

**Authors:** Marco Govoni, Leonardo Vivarelli, Nicola Fazio, Federico Bolognesi, Viscardo Paolo Fabbri, Alessandra Maso, Elisa Storni, Giulia Querzoli, Deyanira Contartese, Stefania Pagani, Luca Cavazza, Marta Pluchino, Lucia De Franceschi, Gianluca Giavaresi, Dante Dallari

**Affiliations:** 1 Reconstructive Orthopaedic Surgery and Innovative Techniques—Musculoskeletal Tissue Bank, IRCCS Istituto Ortopedico Rizzoli, 40136 Bologna, Italy; leonardo.vivarelli@ior.it (L.V.); marta.pluchino@ior.it (M.P.); lucia.defranceschi@ior.it (L.D.F.); dante.dallari@ior.it (D.D.); 2 Technology Transfer Office, IRCCS Istituto Ortopedico Rizzoli, 40136 Bologna, Italy; nicola.fazio@ior.it; 3 Maxillofacial Surgery Unit, Head and Neck Department, San Paolo University Hospital, University of Milan, 20142 Milan, Italy; federico.bolognesi@unimi.it; 4 Department of Laboratory Medicine and Pathology, Azienda USL of Modena, 41121 Modena, Italy; fabbri.viscardopaolo@aou.mo.it; 5 Laboratory of Microbiology and GMP Quality Control, IRCCS Istituto Ortopedico Rizzoli, 40136 Bologna, Italy; alessandra.maso@ior.it (A.M.); elisa.storni@ior.it (E.S.); 6 Pathology Unit, IRCCS Azienda Ospedaliero Universitaria di Bologna, 40138 Bologna, Italy; 7 Department of Medical and Surgical Sciences (DIMEC), University of Bologna, 40138 Bologna, Italy; 8 Surgical Sciences and Technologies, IRCCS Istituto Ortopedico Rizzoli, 40136 Bologna, Italy; deyanira.contartese@ior.it (D.C.); stefania.pagani@ior.it (S.P.); luca.cavazza@ior.it (L.C.); gianluca.giavaresi@ior.it (G.G.)

**Keywords:** acellular allogeneic nerve, allogeneic nerve transplantation, decellularisation, ISO Class A cleanroom protocol

## Abstract

Peripheral nerve injuries affect over one million individuals annually worldwide due to various causes such as trauma, metabolic disorders, and autoimmune diseases. While autologous nerve grafting remains the gold standard for treating large-gap nerve injuries, its limitations, including limited tissue availability, donor site morbidity, infection risk, and suboptimal functional recovery, have spurred interest in alternative approaches. Among these, allogeneic nerve grafting has emerged as a promising option, offering structural and functional advantages due to the native architecture of donor nerves. However, immune rejection due to histocompatibility antigens remains a significant challenge. Decellularisation protocols utilising mild detergents have shown the most promise in preserving the extracellular matrix’s structural and regenerative properties while mitigating immunogenicity. The study aimed to adapt and validate a decellularisation protocol for human nerves within our tissue bank, adhering to European and national regulatory guidelines. The protocol, based on the cleanroom-compliant method previously developed by our group, was optimised to reduce tissue handling time and ensure regulatory compliance. Decellularised sural nerves were assessed for extracellular matrix preservation and sterility using European (EU) Pharmacopoeia and European Directorate for the Quality of Medicines & HealthCare (EDQM) guidelines. The results demonstrated the feasibility of producing high-quality acellular nerve allografts (ANAs) that are suitable for peripheral nerve repair, paving the way for cost-effective and widely accessible grafting solutions.

## 1. Introduction

It has been estimated that more than one million people worldwide are affected annually by peripheral nerve injuries from different causes, such as trauma, metabolic or autoimmune diseases, narrowing of the arteries, hormonal imbalances, and tumours [1,2].

In large-gap nerve injuries, although several surgical techniques have been reported with varying success rates, autologous nerve grafting remains the gold standard for bridging the two ends of the damaged nerve [3]. Nevertheless, the limited amount of available tissue, increased donor site morbidity, and increased risk of infection hamper the widespread use of autografts in nerve reconstruction procedures [4]. Interestingly, some earlier studies have reported that only about 50% of patients who undergo autologous nerve transplants regain a good level of function [5,6,7]. As a result, surgeons frequently opt in favour of surgical alternatives, such as the use of synthetic or biologic nerve guidance conduits [8], the tubulisation with autologous veins [9], and end-to-side neurorrhaphy [10].

Among the available nerve grafting alternatives, the use of nerves from deceased donors has emerged as a promising approach in the field of peripheral nerve surgery [11] due to the availability and relative abundance of the tissue, the provision of adequate structural guidance, the natural 3D architecture, length, and calibre suitable for peripheral nerve regeneration [12]. However, the main concern regarding allograft transplantation is represented by immune tolerance, due to histocompatibility antigens present in Schwann cells, the myelin sheath, and interstitial cells. Over the past decades, different procedures—e.g., freezing, gamma radiation, or chemical methods—have been developed to address immune rejection. Although research into improving nerve allograft outcomes is ongoing, the most promising results, in terms of elimination of the immunogenic antigens while preserving the structural and pro-regenerative components of the extracellular matrix (ECM), come from decellularisation protocols based on the use of mild detergents. Currently, in the USA, the Avance^®^ (AxoGen, Alachua, FL, USA) [13] nerve graft is the only FDA-approved commercial nerve allograft intended for the surgical repair of peripheral nerve discontinuities. In Europe, Axogen^®^ is permitted to distribute nerve grafts through tissue banks, which must obtain import approval from the competent national authorities responsible for human tissue.

However, processing nerves within tissue banks equipped with a Good Manufacturing Practice (GMP)-ISO Class A cleanroom environment could significantly reduce the costs associated with nerve purchase and cross-border shipment, thereby improving the accessibility of nerve graft transplantation. Moreover, while available commercial acellular nerve allografts (ANAs) are typically subjected to terminal gamma-ray sterilisation, aseptic tissue preparation in ISO Class A cleanrooms eliminates the need for irradiation, the effects of which on biological and mechanical tissue are still controversial [14,15,16], further reducing costs.

Therefore, the development of an efficient and cost-effective protocol for the decellularisation of human nerves is justified, as it addresses the limitations associated with autografts and standard allografts, while promoting effective nerve repair and minimising the risk of rejection. Although many protocols have been developed for animal nerve sources, both in vitro [17,18,19] and in vivo [20,21], relatively few published methods to date describe an effective decellularisation of human nerves [22,23]. To our knowledge, only the work of Bolognesi et al. [24], based on the previous ex vivo study by Boriani et al. [25], was designed as an ISO Class A cleanroom-compliant method. Their approach minimises tissue handling time within the sterile environment to under 5 h, making it particularly suitable for common aseptic working sessions.

This study aimed at verifying the feasibility of the abovementioned decellularisation technique within our tissue bank to establish and validate an efficient and reproducible methodology for obtaining and distributing cadaver donor ANAs, in accordance with current European and national legislation and guidelines [26,27].

To achieve this, the entire process—from the tissue procurement to storage—was finely tuned. The protocol developed by Bolognesi et al. [24] was slightly modified to ensure compliance with the strict regulations governing tissue banks. Accordingly, we evaluated the efficiency of the modified decellularisation method on sural nerves collected from cadaver donors by maintening ECM proteins in nerve matrix, quantifying cell membrane remnants, and confirming the absence of microbial contamination, in line with the tests outlined in the EU XI Pharmacopoeia and the fifth edition of European Directorate for the Quality of Medicines & HealthCare (EDQM) guidelines [26].

## 2. Results

### 2.1. Microbiological Testing

Microbiological analyses, performed using both standard and automated methods, detected no microbial growth in each tested tissue sample, showing the maintenance of sterility throughout the procurement and processing phases of sural nerves.

### 2.2. Histological Analysis

**Figure 1:**
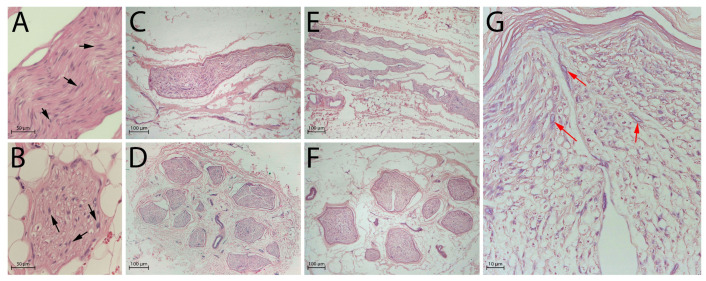
Global nerve preservation evaluated by Haematoxylin and Eosin (H&E) staining. Black arrows indicate intact nuclei in native nerves, red arrows indicate nuclear debris in decellularised nerves: (**A**) native nerve, longitudinal section; (**B**) native nerve, transversal section; (**C**) decellularised nerve after 3 days of freezing, longitudinal section; (**D**) decellularised nerve after 3 days of freezing, transversal section; (**E**) decellularised nerve after 14 days of freezing, longitudinal section; (**F**) decellularised nerve after 14 days of freezing, transversal section; (**G**) high-magnification view of panel (**F**) showing residual nuclear debris (red arrows). Scale bars = 50 µm (**A**,**B**), 100 µm (**C**–**F**), 10 µm (**G**).

Overall nerve structure was evaluated with Haematoxylin and Eosin (H&E) staining (Figure 1A–G).

Native nerves (Figure 1A,B) consisted of three distinct and well-preserved compartments: endoneurium composed of axons and their accompanying Schwann cells; perineurium consisting of concentrically disposed flattened and polygonal cells separated by a thin layer of collagen; epineurium composed of fibrous and adipose tissue with small arteries, veins, and lymphatic vessels.

After decellularisation, the epineurium and the perineurium fibrous tissue were well-preserved in both experimental groups (Figure 1C,D: processing after 3 days of freezing; Figure 1E,F: processing after 14 days of freezing), but no intact cell nuclei were detectable in these regions.

Structures of endoneurium were generally preserved, but a small number of degenerated nuclei and residual chromatin debris were still noticeable within this compartment (Figure 1G).

**Figure 2:**
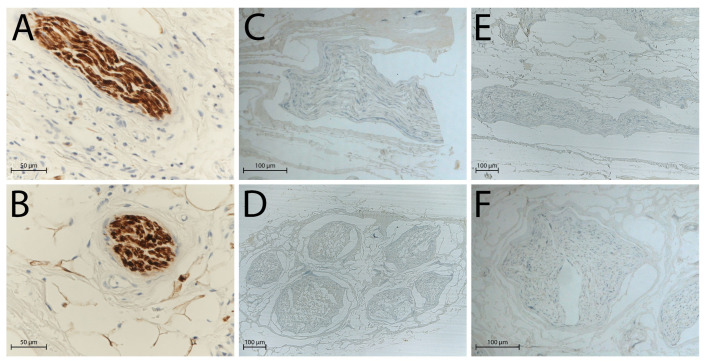
Schwann cells evaluated by S100 antibody, highlighting the absence of immunopositivity in decellularised nerves: (**A**) native nerve, longitudinal section; (**B**) native nerve, transversal section; (**C**) decellularised nerve after 3 days of freezing, longitudinal section; (**D**) decellularised nerve after 3 days of freezing, transversal section; (**E**) decellularised nerve after 14 days of freezing, longitudinal section; (**F**) decellularised nerve after 14 days of freezing, transversal section. Scale bars = 50 µm (**A**,**B**), 100 µm (**C**–**F**).

**Figure 3:**
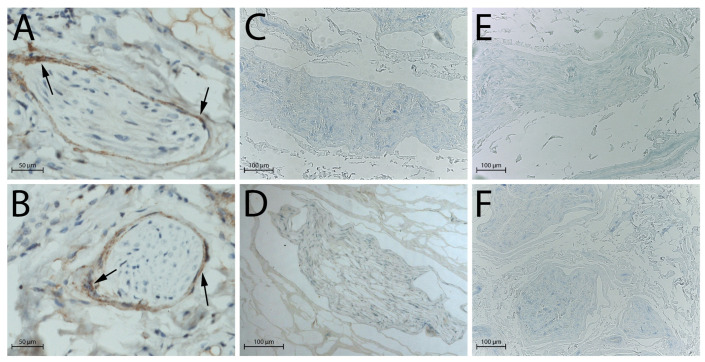
Perineurial cells evaluated by EMA antibody, black arrows indicate the presence of perineurial cells in native nerves, absents in decellularised nerves: (**A**) native nerve, longitudinal section; (**B**) native nerve, transversal section; (**C**) decellularised nerve after 3 days of freezing, longitudinal section; (**D**) decellularised nerve after 3 days of freezing, transversal section; (**E**) decellularised nerve after 14 days of freezing, longitudinal section; (**F**) decellularised nerve after 14 days of freezing, transversal section. Scale bars = 50 µm (**A**,**B**), 100 µm (**C**–**F**).

S100 and epithelial membrane antigen (EMA) immunohistochemical staining confirmed these observations: in native nerves, Schwann cells (Figure 2A,B) and perineurial cells (Figure 3A,B) were clearly identifiable through strong immunoreactivity, whereas after decellularisation, no distinct immunopositivity was observed in either group (Figure 2C,D and Figure 3C,D: processing after 3 days of freezing; Figure 2E,F and Figure 3E,F: processing after 14 days of freezing).

**Figure 4:**
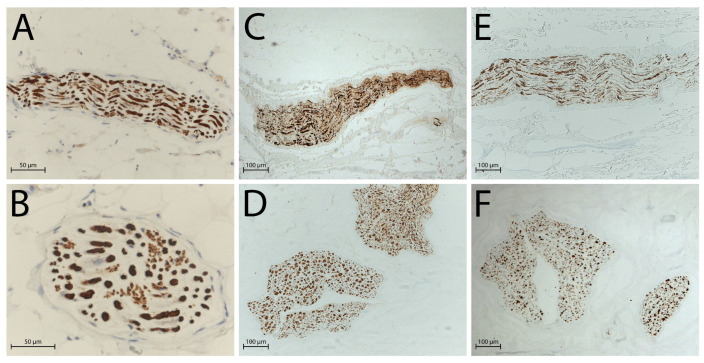
Axonal network evaluated by NF antibody, with a global preservation of network in decellularised nerves: (**A**) native nerve, longitudinal section; (**B**) native nerve, transversal section; (**C**) decellularised nerve after 3 days of freezing, longitudinal section; (**D**) decellularised nerve after 3 days of freezing, transversal section; (**E**) decellularised nerve after 14 days of freezing, longitudinal section; (**F**) decellularised nerve after 14 days of freezing, transversal section. Scale bars = 50 µm (**A**,**B**), 100 µm (**C**–**F**).

**Figure 5:**
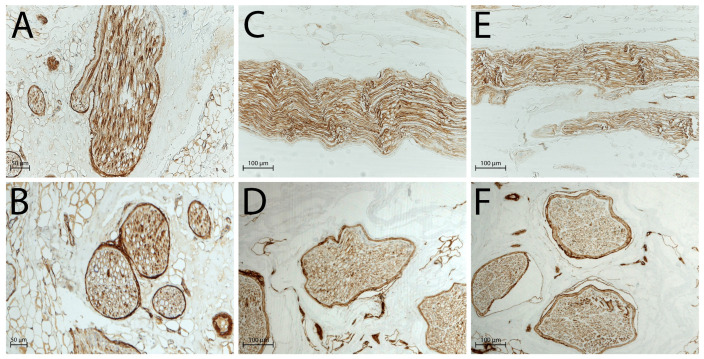
ECM component evaluated by type IV collagen antibody, evidencing a preservation of the matrix: (**A**) native nerve, longitudinal section; (**B**) native nerve, transversal section; (**C**) decellularised nerve after 3 days of freezing, longitudinal section; (**D**) decellularised nerve after 3 days of freezing, transversal section; (**E**) decellularised nerve after 14 days of freezing, longitudinal section; (**F**) decellularised nerve after 14 days of freezing, transversal section. Scale bars = 50 µm (**A**,**B**), 100 µm (**C**–**F**).

Native nerves contained well-preserved axons and ECM, whereas the decellularised group retained moderately preserved axonal proteins and well-preserved ECM components, as confirmed by the immunohistochemical staining for neurofilament (NF; Figure 4) and type IV collagen (Figure 5).

Significant differences were not noticed between specimens processed with different frozen point times.

Histological data are presented in Table 1 and Table 2.

### 2.3. DAPI Staining

**Figure 6:**
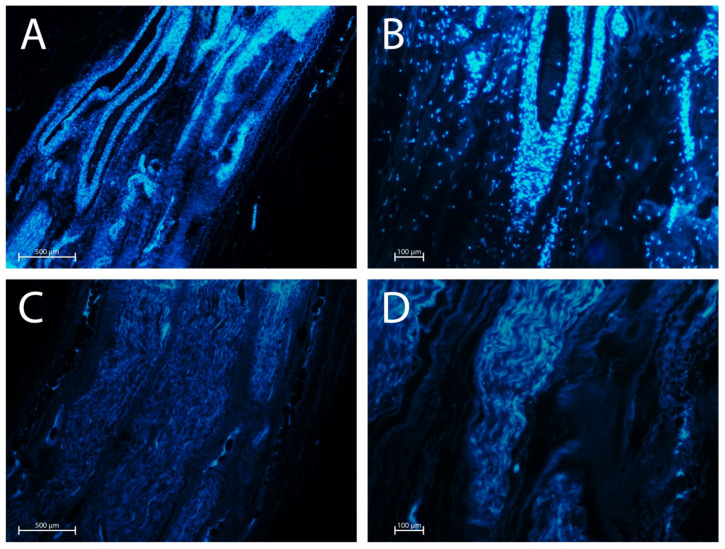
DAPI staining of native and decellularised nerves to reveal nuclei (magnification 4×, (**A**,**C**); magnification 10×, (**B**,**D**)). (**A**,**B**) Native nerve samples; (**C**,**D**) decellularised nerve samples. Scale bars = 500 µm (**A**,**C**), 100 µm (**B**,**D**).

Assessment of nuclear count using DAPI staining showed that the decellularisation protocol resulted in a significantly reduced number of cells compared to native nerves (Figure 6).

## 3. Discussion

Although autologous nerve grafting remains the most common approach for repairing peripheral nerve injuries, there is growing interest in alternative treatment strategies. This shift is largely driven by the disadvantages associated with autografts, including donor site morbidity, loss of function, and increased infection risk, which make this procedure less attractive to both surgeons and patients.

Therefore, developing any surgical methods that enhance functional recovery following peripheral nerve lesion or rupture would offer significant benefits to healthcare systems and society as a whole.

Commercial artificial nerve conduits available on the market represent a valid alternative to autografts. As an example, NeuraGen^®^ (Integra LifeSciences, Princeton, NJ, USA), a synthetic conduit consisting of purified bovine type I collagen, has demonstrated the ability to support nerve regeneration in various animal models. However, definitive clinical data remain limited. Additionally, Whitlock et al. [28] reported performance limitations of this product in nerve gaps exceeding the critical length of 10 mm.

ANAs represent another promising alternative to autografts. ANAs are processed using different techniques designed to preserve the nerve’s internal structure and ECM proteins; these biological features play a crucial role in guiding nerve fibre elongation during the regeneration process. Moreover, optimal decellularisation protocols have to ensure the complete removal of living cells and/or cellular debris, as their presence may trigger an immune rejection response in the recipient. Over the years, researchers have developed various methods to address this challenge, involving different thermal, physical, and chemical treatments—often used in different combinations—to achieve effective decellularisation. Although all of these techniques are theoretically capable of eliminating cells without damaging the tissue morphology, thermal and physical (i.e., irradiation) treatments often struggle to remove cellular remnants trapped inside the basal laminae tubes, which constitute the structure of the ECM. The presence of degenerated cell material is thought to interfere with the regenerative process promoted by the Schwann cells and macrophages of the host, which begin to invade the basal laminae’s tubes during the first days after the ANA’s implantation [29]. On the other hand, chemical treatments excel in removing cellular material from the allograft, but they tend to be more aggressive than the other methods and risk damaging the ECM structure, especially at high concentrations for extended incubation times.

Currently, the manufacturing of the only ANA commercially available on the market—i.e., Avance^®^—relies on a chemical decellularisation protocol based on the method developed by Hudson et al. [30]. While this approach effectively eliminates cellular material, it may also compromise the biomechanical and biochemical integrity of the graft by disrupting ECM components. This highlights the need for milder detergents that strike an optimal balance between cellular removal and ECM preservation. In this regard, some authors [22,23,24] have opted for Triton X-100 over Triton X-200 since non-ionic agents are relatively milder when compared to their anionic counterparts, as they disrupt the lipid−lipid and lipid−protein interactions [31].

In this study, we present a decellularisation protocol that, while relying on the use of detergents, has been meticulously tuned to comply with the strict guidelines and mandatory ISO standards for European tissue banks. These standards encompass a wide range of requirements, including acceptable time frames for tissue recovery after donor death, strategies to minimise the risks of disease transmission in transplanted allografts, proper storage conditions, rigorous requirements for microbial testing, robust tissue and material traceability protocols, and well-defined procedures and precautions to safeguard cleanroom personnel, equipment, and infrastructure [26]. Adherence to these standards is imperative for tissue banks to achieve certification by the relevant authorities.

Regarding the specific technical aspects of our decellularisation procedure, we have introduced some minor modifications to the detergents employed for the allograft processing compared to previously established protocols. It is worth noting that in 2020, the European Chemical Agency included the principal component of Triton X-100—i.e, 2-[4-(2,4,4-trimethylpentan-2-yl)phenoxy]ethanol—on the Registration, Evaluation, Authorization, and Restriction of Chemicals (REACH) list of substances with potential endocrine-disrupting properties [32].

However, although the sunset date for Triton X-100 commercialisation was set in January 2021, exemptions to the REACH regulation exist for its use in medical, veterinary, alimentary, and cosmetic products. Therefore, pharmaceutical industries may continue using Triton X-100, without prior notification or authorisation, provided that the final concentration in the end product does not exceed 0.1% (*w*/*w*).

Nevertheless, to ensure high levels of safety for the patients and to mitigate the risk of future restrictions on the use of Triton X-100, we opted to replace it with Tween^®^ 20, a comparable alternative. Although both detergents are commonly used for permeabilising cell membranes, Tween^®^ 20 is the milder of the two.

Following EDQM’s requirement and the guidelines of the Italian National Transplant Centre (Centro Nazionale Trapianti, CNT), biopsies collected during the procurement must test negative for microbial contamination before the tissue can enter the cleanroom. Therefore, the rationale for using two different freezing times is aligned with EDQM’s requirement. When the direct inoculation method is performed, samples must be incubated for up to 14 days; on the other hand, if alternative microbiological testing methods offering automated, rapid, and more sensitive microbiological results are used, the incubation period is reduced to 7 days. Accordingly, depending on the available equipment of a tissue bank, this protocol offers two options: 5 days of gentle agitation followed by 3 days of freezing or 14 days of direct storage at −80 °C. This flexibility ensures compatibility with both standard and alternative methods of microbial testing.

DAPI staining confirmed the presence of intact cell nuclei, appreciable as blue fluorescent dots (cell nuclei) in native nerves. In contrast, decellularised nerves exhibited no strong fluorescence, with a marked reduction in DAPI staining, indicating effective removal of cellular components. This result was confirmed by histopathological analysis with H&E staining and immunohistochemical evaluations, which also showed the satisfactory preservation of the global nerve structure and the ECM. Endoneurium, perineurium, and epineurium remained well-preserved, demonstrating the structural integrity necessary for potential regenerative processes. This result underscores the ability of the protocol to maintain the architectural fidelity of the decellularised nerves, which is pivotal for successful integration and functionality in clinical applications.

Specifically, the H&E staining revealed that, although no intact nucleus or cytoplasmic structure of Schwann cells was detectable, the basal lamina of the ECM showed continuity and structural stability. This finding is significant, as the ECM provides essential guidance for axonal regrowth, including biochemical and mechanical signals, and provides a scaffold for cellular infiltration during nerve repair [33]. The preservation of these structural elements ensures that the microenvironment required for Schwann cell migration and axonal sprouting remains physiologically functional, facilitating the initial phases of nerve regeneration post-implantation [34].

Immunohistochemistry confirmed these findings: the axonal network, marked by NF staining, showed moderate conservation, with recognizable filamentous structures that retained some degree of organization and alignment. This level of axonal integrity, albeit reduced compared to native nerves, should be sufficient to support regeneration once host-derived axons begin to infiltrate the graft. As reported by some studies [30,35,36,37], partial retention of structured NFs could provide a beneficial scaffold for regenerating axons by preserving the original axonal pathways and architecture, maintaining molecular cues or binding sites that facilitate directed regrowth, and supporting the alignment of Schwann cells once repopulated, which is key for remyelination.

The absence of viable Schwann cells, as indicated by the lack of immunopositivity of S100 and EMA, prepares the field for the necessary host-derived cell contribution. These host cells, including endogenous Schwann cells and macrophages, will play a crucial role in degrading residual debris, remodelling the ECM, and promoting axonal growth during the regeneration process. The complete nuclear degeneration of Schwann cells was also confirmed in two cases by anti-SOX10 antibody, a transcription factor present in the nuclei of all neuroectodermal cells, which gave a negative result (Figure S1).

The structural and compositional integrity of the ECM was further supported by the bright and continuous immunoreactivity test, which was positive for type IV collagen staining, underscoring the successful preservation of key ECM proteins essential for the regenerative microenvironment. The strong presence of type IV collagen is critically important in supporting Schwann cell adhesion and proliferation, enhancing the overall regenerative potential of the graft [38]. Moreover, type IV collagen forms a supportive substrate that not only stabilizes the architecture of the scaffold but also interacts with integrin receptors on growing axons, facilitating directed outgrowth. Specifically, type IV collagen contributes to the formation of aligned ECM channels that can serve as conduits for axonal extension [39,40,41].

Interestingly, comparative evaluations (Table 1 and Table 2) revealed no significant difference in tissue preservation between the two freezing protocols tested (3 days vs. 14 days at −80 °C). This finding indicates that the protocol is resilient to variations in freezing durations, offering flexibility in its implementation across different tissue bank workflows. Such adaptability enhances the protocol’s practicality for large-scale applications, ensuring consistency in graft quality regardless of logistical constraints. EuroGTP II—an interactive assessment tool developed by EDQM for identifying, quantifying, and assessing the level of risk associated with novel substances of human origin (SoHO) preparation [42,43]—defined the TCTPs (Tissue and Cells Therapies and Products; i.e., acellular nerve allograft) prepared within our tissue bank as safe, efficacious for clinical use, and very unlikely to cause harm to recipients.

Taken together, the presented results validate the efficacy of the decellularisation protocol in preserving key structural and molecular components of the nerve while eliminating immunogenic cellular material. These characteristics make nerves treated by the present method suitable for use as acellular nerve allografts in the clinical setting, supporting both functional integration and regenerative potential.

It is worth noting that, in the present protocol, allogeneic nerves have not undergone enzymatic degradation of chondroitin sulphate proteoglycans (CSPGs), despite evidence from other studies indicating that the CSPG degradation markedly enhances the axonal regeneration [44,45,46]. However, these studies involved allogenic nerves harvested from adult rats and subject to enzymatic degradation coupled with a mild thermal processing. Therefore, such results are not directly comparable to outcomes from detergent-based protocols.

Currently, only Avance^®^ nerve grafts are processed using a combination of detergent-based decellularisation followed by enzymatic degradation [47]. Nevertheless, there are no data in the literature that demonstrate the superiority of Avance^®^ nerve grafts, in terms of nerve regeneration, compared to other processed human nerve grafts due to the lack of a similar available product on the market. Most available published scientific papers are either retrospective studies [48,49,50] or research articles comparing Avance^®^ products with synthetic conduits [28,51].

Indeed, the goal of the presented protocol is to introduce the first available alternative to commercial ANAs for clinical use to the scientific community. Table 3 summarizes the state of the art about available protocols for the decellularisation of allogeneic nerves, focusing on chemical/enzymatic/physical treatments, protocol duration, regulatory compliance, and clinical readiness level.

Notably, Ansaripour et al. [52] have implemented a deterministic and probabilistic cost-effectiveness analysis for the use of Avance^®^ allograft compared with autograft in patients who are candidates for nerve reconstruction in the USA, showing a high probability of saving costs and being more effective over a lifetime horizon.

Likewise, theoretically applying the same considerations to Europe and taking into account that, compared to Avance^®^, our products do not need a terminal sterilisation by gamma rays, further cost reduction is expected. Based on preliminary evaluations, we estimate a cost reduction of approximately 30% to 60%, depending on the length of the nerve segment, compared to Avance^®^. However, the final cost can only be determined upon completion of clinical trials and the initiation of routine clinical distribution of the tissue.

While the presented decellularisation protocol for ANAs shows promising results, complies with current regulatory standards, and has received approval from National Authorities, some limitations should be acknowledged and warrant further investigation.

First, although previous studies in rabbits have reported no signs of pain, discomfort, clinical lesions, or graft rejection—and have shown comparable conduction velocities between autografts and ANAs [25,53]—these findings still require further evaluation in clinical settings, especially regarding regeneration rate, conduction recovery, and behavioural outcomes in patients. Moreover, as immune responses to allogeneic nerve grafts are a major factor contributing to graft failure, the ability to accurately quantify anti-donor immunity is a critical aspect of peripheral nerve transplantation. Such immunological assessments could be implemented in clinical settings to help predict recipient pre-sensitisation to the graft and enable early detection of rejection, potentially reducing the need for invasive procedures and improving clinical outcomes [54]. However, these assays are costly and difficult to perform in most clinics or tissue banks. A common strategy to minimise the risk of immune rejection in decellularised tissues involves the use of DNase to reduce residual host genomic DNA. Nevertheless, while DNase treatment can effectively lower gDNA content, it may also compromise tissue integrity by disrupting ECM architecture, potentially affecting the mechanical and biological properties of the graft. This highlights the need to carefully balance DNA removal with ECM preservation [55], and further studies are necessary to address this challenge.

Second, the long-term storage stability of the processed grafts remains to be fully characterized. Systematic evaluation of different storage durations is needed to establish protocols that preserve both the structural and functional integrity of the grafts over time.

Third, multicentric validation across different tissue banks and clinical environments is essential to confirm the reproducibility, generalizability, and adaptability of the protocol to varied operational workflows and patient populations. In this context, the incorporation of quantitative analyses (e.g., ELISA, Western blotting), as opposed to relying solely on qualitative techniques, could provide precise information on protein expression levels in the ANAs, thereby supporting improved standardisation in their production.

Finally, potential future improvements to the presented decellularisation process may include the following: (i) an enhanced DNA removal technique through an optimally balanced combination of a mild enzymatic digestion and physical methods such as sonication or perfusion-based washing, which may further reduce residual DNA without compromising ECM integrity; (ii) an optimisation of detergent concentrations and exposure time to improve cell removal efficiency while preserving ECM components critical for nerve regeneration; (iii) a fine-scale monitoring of the decellularisation progress, using advanced monitoring techniques, such as high-resolution imaging [56] and molecular biomarkers [57], which could enhance quality control by enabling non-invasive assessment of decellularisation efficiency and ECM preservation.

## 4. Materials and Methods

### 4.1. Preparation of P-Nerve and D-Nerve Sterile Solutions

P-Nerve and D-Nerve solutions are used during the tissue procurement and cleanroom sample decellularisation, respectively.

P-Nerve consists of 50 mL of sterile saline with 125 mM caprylyl sulfobetaine (SB3-10; Merck, Darmstadt, Germany) and 0.2% *v*/*v* Tween^®^ 20 (Merck), while D-Nerve contains 0.25% *w*/*v* sodium dodecyl sulfate (SDS; Merck) in 200 mL of sterile saline. Both solutions are filtered by 0.20 μm sterile filters and stored at −20 °C until use. Moreover, to guarantee the absence of microbial contamination, each solution is tested with the BACTEC^TM^ FX automated system (Becton Dickinson Spa, Florence, Italy), an instrument with advanced fluorescence detection technology for the detection of microbial species, as detailed below.

### 4.2. Donor Selection and Tissue Collection

Nerve procurement and processing were performed by the Musculoskeletal Tissue Bank of IRCCS Istituto Ortopedico Rizzoli (Bologna, Italy; EU TE Code: IT000096) [58,59], a public non-profit organization authorized by the Italian National Transplant Centre (Centro Nazionale Trapianti, CNT) for the procurement, processing, storing, and distribution of human musculoskeletal tissue, and recently, acellular allogeneic nerves.

As previously reported [60], serological tests were performed on all selected donors to exclude the transmission of hepatitis, T-cell lymphotropic virus, or immunodeficiency viral infections, and individual travel history in a high-risk region for endemic viruses was evaluated to perform potentially required additional tests.

**Figure 7:**
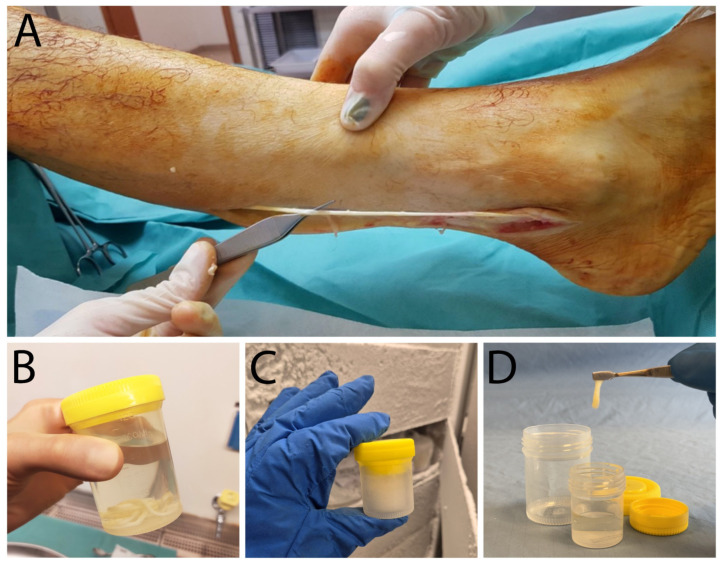
(**A**) Surgical procedure for sural nerve procurement. (**B**) Nerve segment inserted in a sterile container and immersed in the P-Nerve sterile solution with an antibiotic cocktail. (**C**) Nerve segment inserted in a sterile container, immersed in sterile saline solution, and stored at −80 °C. (**D**) Thawed nerve segment ready to use.

Three right leg sural nerves were collected aseptically from three different cadaver donors (mean age: 55 years; 2 females, 1 male) within 24 h post-mortem. Nerves were dissected, and the perineural adipose tissue was removed. From each nerve, two segments of about 15 cm in length and 0.25 cm in diameter were obtained (Figure 7A), immediately inserted in two different sterile containers, and immersed in the P-Nerve sterile solution added in the operating room with an antibiotic cocktail (Figure 7B) containing 0.05 mg/mL vancomycin hydrochloride (Fisiopharma S.r.l., Salerno, Italy), 1000 IU/mL ceftazidime (Biopharma S.r.l., Rome, Italy), and 0.33 mg/mL colistimethate sodium (Accord Healthcare S.L.U., Barcelona, Spain). After the procurement, samples were incubated for 120 h at room temperature (RT) on the Advanced 3500 digital shaker (VWR International S.r.l., Milan, Italy), placed in an ISO Class D environment, and then frozen at −80 °C until their processing within the ISO Class A cleanroom environment.

### 4.3. Decellularisation Protocol

To perform the decellularisation protocol steps within an ISO Class A cleanroom environment, it is mandatory that incoming samples test negative for aerobic/anaerobic bacteria and fungi. According to EDQM guidelines, incubation conditions for sample sterility testing have to be performed in a testing period of 7 or 14 days, depending on whether microbiological analysis methods are carried out by automated culture systems or direct inoculation methods, respectively. In this regard, we have subjected samples to freezing up to 3 or 14 days—after their agitation on an orbital shaker for 120 h at RT—with the aim to make this protocol suitable for European tissue banks which perform microbiological testing by standard, automated, or both methods. Moreover, the same protocol may also be useful for all the tissue banks worldwide that apply similar guidelines.

Opening the containers in the cleanroom marks the start of the 5-h direct nerve manipulation process. Steps of the decellularisation protocol are listed as follows:

− Samples and the D-Nerve solution are thawed at 4 °C overnight.
− The day after, inside an ISO Class A cleanroom environment, the P-Nerve solution is discarded, and thawed nerves are placed on a sterile stainless-steel board and cut with a sterile scalpel blade in segments of required dimensions.
− All the segments are inserted in a sterile polypropylene/polyethylene (PP/PE) container (Agricons Ricerche, Padua, Italy), immersed in 50 mL of sterile saline solution, and agitated with the incubator shaker MD3000 (PBI S.r.l., Milan, Italy) for 10 min at RT. This washing step is repeated three times with fresh sterile saline solution.
− Each segment is inserted in a sterile 50 mL tube, immersed in about 30 mL of D-Nerve solution, and undergoes five cycles of agitation/sonication at RT. Agitation steps are performed with the MD3000 incubator shaker (PBI-International, Milan, Italy) for 27 min, while ultrasound irradiation is carried out by the Sonica 3200L ultrasonic bath (SOLTEC S.r.l., Milan, Italy), featuring a stainless steel tank and autoclavable accessories, at the frequency of 39 kHz ± 1 kHz with SWEEP SYSTEM Technology for 3 min. Specifically, the SWEEP SYSTEM technology adjusts the amplitude of the electrical signal with an oscillating frequency of 39 kHz ± 1 kHz, enhancing ultrasonic cavitation for superior performance [61].
− Three further washing steps (10 min each) with sterile saline are needed to conclude the decellularisation protocol.
− ANAs are then immersed in sterile saline solution inside PP/PE sterile containers (Agricons Ricerche) and stored at −80 °C (Figure 7C) until their use (Figure 7D).

### 4.4. Microbiological Tests

During the tissue procurement activities, the entire surface of nerves is swabbed using sterile swab sticks (Copan, Murrieta, CA, USA). The tubes containing swab sticks immersed in the liquid Amies preservation medium are then vigorously vortexed by a vortex mixer, and the media are inoculated in Thioglycolate Medium and Saboraud broth (Biolife Italiana, Milan, Italy) and cultured for 14 days to exclude the presence of anaerobic bacteria and yeasts or fungi, respectively. Moreover, to determine the potential presence of aerobic bacteria, a representative sample of each nerve entering the bank is cultured in Tryptic Soy broth (Biolife Italiana) for the same time.

At the end of the decellularisation protocol in cleanroom, 10 mL of the final sample washing solution is (i) injected into BD BACTEC^TM^ culture bottles (Becton Dickinson) containing patented resins capable of effectively inactivating antibiotics and then (ii) analysed by the BACTEC^TM^ FX automated system for 7 days to assess the absence of microbial contamination after the decellularisation process.

### 4.5. Histological Analysis

Native and decellularised nerves were cut into three cross-sections (surgical margins with a central portion) and two longitudinal sections. Fragments were fixed in 4% (*v*/*v*) phosphate-buffered paraformaldehyde and paraffin-embedded for light microscopy and immunohistochemistry. Then, 5 µm-thick sections were mounted on a slide (dewaxed, rehydrated, and prepared for different stains). Routine staining with H&E (Sigma-Aldrich S.r.l., Milan, Italy) was performed to evaluate general morphology and the presence of cell nuclei.

Immunohistochemistry was performed on an automated stainer, using Ventana-purchased pre-diluted antibodies (Ventana, Tucson, AZ, USA), according to standardised protocols: anti-S100 (polyclonal) highlighted Schwann cells, anti-EMA (epithelial membrane antigen, clone E29) was used for perineurium, anti-NF (neurofilament, clone 2F11) demonstrated axons, and anti-type IV collagen (clone CIV22) stained the ECM of the nerve.

To compare the decellularisation outcomes, the following scores were adopted to assess the different nerve histological and immunohistochemical characteristics:

(a) For endoneurium, perineurium, and epineurium preservation (global nerve preservation): badly preserved, moderately preserved, well preserved;

(b) For nuclear density: absent = 0, low < 25/High-Powered Field (HPF), medium = 25–50/HPF, high > 50/HPF;

(c) For nuclear status: intact or degenerated (nuclear debris without distinct shape);

(d) For Schwann Cells density (distinct immunopositivity for S-100 both in the nucleus and cytoplasm): absent = 0, low < 25/HPF, medium = 25–50/HPF, high > 50/HPF;

(e) For perineurial cells density (distinct membrane immunopositivity for EMA): absent = 0; low < 25/10 High-Powered Field (HPF); medium = 25–50/HPF; high > 50/HPF;

(f) For axonal network: well-preserved (intense immunostaining, preserved shape, parallel orientation for NF), moderately preserved (axonal swelling but strong immunointensity), badly preserved (reduced immunointensity for NF and axonal swelling);

(g) For ECM proteins: well-preserved (strong, continuous, and homogeneous immunointensity for type IV collagen) or badly preserved (weak and heterogeneous immunoreactivity).

### 4.6. DAPI Fluorescence

Samples of native and decellularised nerves were fixed in 10% formalin and embedded in paraffin. Histological sections, cut to a thickness of 5 µm with a microtome, were stained with 4′,6-diamidino-2-phenylindole (DAPI; Invitrogen SRL, San Giuliano Milanese, MI, Italy) to fluorescently label the nucleic acids. After staining, the samples were examined using an Olympus IX71 fluorescence microscope (Olympus, Tokyo, Japan), and images were acquired with an Olympus XC digital image capture system (Olympus).

## 5. Conclusions

In the European Union (EU), the relevant directives outlining the requirements for tissue and cell donation, processing, and distribution, namely EDQM, have been transposed into the national legislation of the EU member states, offering a valuable source of practical guidance for stakeholders operating within the EU legislative framework. In this article, we presented a novel decellularisation protocol based on the use of non-ionic and anionic detergents, specifically tuned for ISO Class-A cleanroom environments following EDQM guidelines and approved by Italian Competent Authorities in November 2023.

Nevertheless, while the protocol demonstrates structural feasibility and regulatory readiness for clinical translation, its functional and immunological performance remains to be fully validated. To this aim, a clinical trial, currently in the recruitment phase, will be essential to assess the protocol’s efficacy in terms of axonal regeneration and functional recovery. However, by sharing this protocol with the international scientific community, we aim to (i) pave the way for the development of feasibility studies carried out by other public/private tissue banks located in Europe or other jurisdictions with comparable regulations, (ii) contribute to expanding the list of products available for the reconstruction of nerve injury, and (iii) support future efforts toward reducing the overall treatment burden associated with peripheral nerve injuries.

## Figures and Tables

**Table 1:** Decellularisation score for specimens after 3 days of freezing at −80 °C.

Sample N.	Global Nerve Preservation	Nuclear Status	Intact Schwann Cells	Intact Perineural Cells	Axonal Network Preservation	ECM Preservation
1	well	degenerated	absent	absent	moderate	well
2	well	degenerated	absent	absent	moderate	well
3	well	degenerated	absent	absent	moderate	well

**Table 2:** Decellularisation score for specimens after 14 days of freezing at −80 °C.

Sample N.	Global Nerve Preservation	Nuclear Status	Intact Schwann Cells	Intact Perineural Cells	Axonal Network Preservation	ECM Preservation
1	well	degenerated	absent	absent	moderate	well
2	well	degenerated	absent	absent	moderate	well
3	well	degenerated	absent	absent	moderate	well

**Table 3:** Comparison of available decellularisation protocols for allogeneic nerves.

Decellularisation Protocol	Chemical Reagents	Enzymatic Treatment	Physical Treatment	Duration	Regulatory Compliance	Clinical Readiness Level
Our protocol	Tween 20 SB3-10 SDS	/	Sonication, washing	∼5 days	Approved by competent authorities	Moderate/high (GMP, EDQM, national approval)
Avance^®^	SB-10 SB-16 NaCl Sodium phosphate buffer Phosphate buffer	CSPGs	Washing, terminal irradiation	>3 days	FDA certified	High (commercially available)
Bae et al. [22]	Triton X-100 NaCl Sodium deoxycholate	/	Washing	∼6 days	Research use only	Low
	CHAPS NaCl	DNase RNase A	Washing	∼5 days	Research use only	Low
Nieto-Nicolau et al. [23]	Triton X-100 SB3-10 SB3-16 Tris-EDTA	DNase	Washing	∼7 days	Research use only	Low

## Data Availability

Data pertaining to histological analyses and nuclear staining are available upon request from the corresponding author.

## Supplementary Materials

The supporting information can be downloaded at https://www.mdpi.com/article/10.3390/ijms26167937/s1.

## Abbreviations

The following abbreviations are used in this manuscript:

ANA	Acellular nerve allograft
DAPI	4′,6-diamidino-2-phenylindole
ECM	Extracellular Matrix
EDQM	European Directorate for the Quality of Medicines & HealthCare
EMA	Epithelial Membrane Antigen
FDA	Food and Drug Administration
GMP	Good Manufacturing Practice
GTP	Good Tissue Practice
H&E	Haematoxylin and Eosin
HPF	High-Powered Field
NF	Neurofilament
PP/PE	Polypropylene/Polyethylene
REACH	Registration, Evaluation, Authorization, and Restriction of Chemicals
SoHO	Substances of Human Origin
RT	Room Temperature
TCTPs	Tissue and Cells Therapies and Products
