# Supplementary material for: Peripheral Nerve Decellularisation Protocol for Allogeneic Transplantation: From Tissue Procurement to Banking
Source: Int J Mol Sci. 2025 Aug 17;26(16):7937. doi: 10.3390/ijms26167937 (PMC12386939; doi:10.3390/ijms26167937)
Supplement: Supplementary file 1 [file ijms-26-07937-s001.zip › Anti-SOX10 staining.pdf]

Anti SOX-10 staining

SOX-10 immunohistochemistry was performed in an automated stainer, using Ventana-purchased pre-diluted antibodies (Ventana, Tucson, AZ, USA), according to standardised protocols. Anti-SOX10 (SOX-10 polyclonal antibody, Cell Marque, Millipore Sigma) was used in two cases for confirmation of nuclear degeneration of Schwann cell.

Figure S1 shows the complete nuclear degeneration of Schwann cells performed in two cases by anti-SOX10 antibody, a transcription factor presents in the nuclei of all neuroectodermal cells, which gave a negative result.

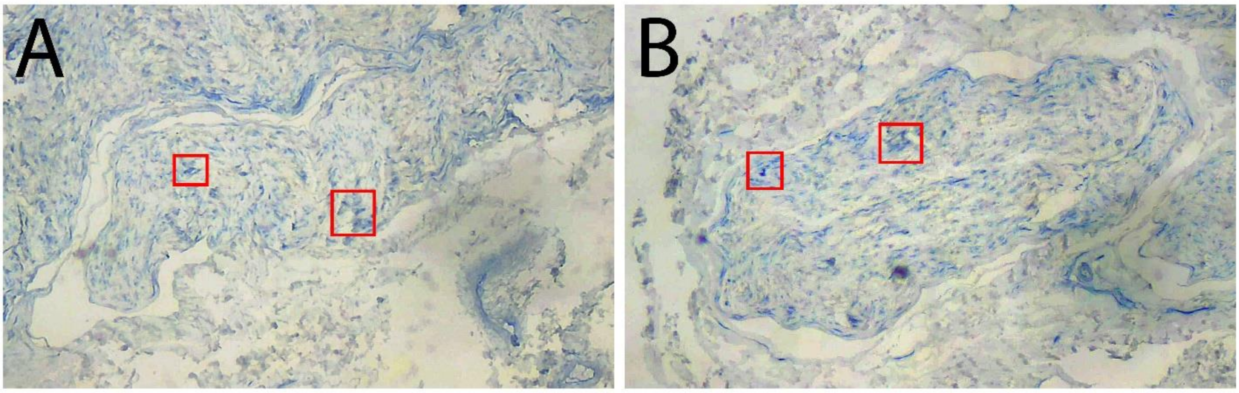

Figure S1. SOX10 antibody was negative in degenerated nuclei of decellularised nerve, red boxes highlight nuclear debris: A) decellularised nerve after 3 days of freezing, longitudinal section; B) decellularised nerve after 14 days of freezing, transversal section.
