# Supplementary material for: Peripheral Nerve Decellularisation Protocol for Allogeneic Transplantation: From Tissue Procurement to Banking
Source: Int J Mol Sci. 2025 Aug 17;26(16):7937. doi: 10.3390/ijms26167937 (PMC12386939; doi:10.3390/ijms26167937)
Supplement: Supplementary file 1 [file ijms-26-07937-s001.zip › ijms-3789385_EuroGTP II.pdf]

EuroGTP II Interactive Assessment Tool

TCTP: *Tissues - Other*

The following information refers to TCTP: *Acellular Nerve*

Evaluation performed on: *2023-06-15 16:09:17*

Description of TCTP under evaluation: *No description has been provided*

	Yes	*No*	*NA*
A. Has this type of TCTP previously been prepared and issued for clinical use by your establishment?		X	
B. Will the starting material used to prepare this TCTP be obtained from the same donor population previously used by your establishment for this type of TCTP?	X		
C. Will the starting material for this TCTP be procured/collected using a procedure used previously by your establishment for this type of TCTP?		X	
D. Will this TCTP be prepared by a procedure (processing, decontamination and preservation) used previously in your establishment for this type of TCTP?		X	
E. Will this TCTP be packaged , stored , and distributed using a protocol and materials used previously in your establishment for this type of TCTP?	X		
F. Will this type of TCTP provided by your establishment be applied/infused clinically using an application/infision method used previously?	X		
G. Has your establishment provided this type of TCTP for a same clinical indication or applied/infused into a same anatomical site?		X	

	Justification provided for Evaluation of Novelty questions
No justification has been provided	

Risk Factor	*Risk*	*Probability*	*Severity*	*Detectability*	*Potential Risk*	*Risk Reduction*	*Risk*
Donor Characteristics	Unwanted immunogenicity	2	2	5	**20**	75%	**5**
Donor Characteristics	Implant failure	3	1	5	**15**	75%	**3.75**
Donor Characteristics	Disease transmission	1	3	2	**6**	95%	**0.3**
Recovery process and environment	Implant failure	2	1	2	**4**	75%	**1**
Recovery process and environment	Disease transmission	1	2	2	**4**	95%	**0.2**
Processing and environment	Implant failure	2	1	3	**6**	75%	**1.5**
Processing and environment	Disease transmission	1	2	2	**4**	95%	**0.2**
Reagents	Toxicity / Carcinogenicity	2	2	5	**20**	75%	**5**
Reliability of Microbiology Testing	Disease transmission	1	2	2	**4**	75%	**1**
Storage conditions	Implant failure	3	1	5	**15**	50%	**7.5**
Transport conditions	Implant failure	1	1	1	**1**	75%	**0.25**
Complexity of the pre-implantation preparation and/or application method	Implant failure	2	1	2	**4**	50%	**2**

Risk Factor	*Applicable*	*Comment*
No comments provided		

Your assessment has Final Risk Score of: **2**

This suggests that your TCTP falls into the Level of Risk:

Level of Risk	*Extent of Studies needed*
Negligible	**Step3A: Risk reduction strategies**<br><br>The assessment indicates that the TCTP is safe and efficacious for clinical use and very unlikely to cause harm to recipients. You should conduct a validation of the process, if not already done. If the nature of the risk is not related to the process itself, the requirement for validation may not apply, for example where the novelty is in the method of clinical application.
	**Step 3B: Extent of clinical evaluation**<br><br>Adverse reaction and event (SARE) reporting.

Please refer to EuroGTP II Guide for additional details.

Print Restart Save
